# Anti-SARS-CoV-2 Immunoglobulins in Human Milk after Coronavirus Disease or Vaccination—Time Frame and Duration of Detection in Human Milk and Factors That Affect Their Titers: A Systematic Review

**DOI:** 10.3390/nu15081905

**Published:** 2023-04-14

**Authors:** Margarita Dimitroglou, Rozeta Sokou, Nicoletta Iacovidou, Abraham Pouliakis, Georgios Kafalidis, Theodora Boutsikou, Zoi Iliodromiti

**Affiliations:** Neonatal Department, Medical School, National and Kapodistrian University of Athens, Aretaieio Hospital, 11528 Athens, Greece

**Keywords:** human milk, COVID-19, SARS-CoV-2 virus, immunoglobulin levels, milk-transferred antibody, breastfeeding, immunology

## Abstract

Human milk (HM) of mothers infected with or vaccinated against SARS-CoV-2 contains specific immunoglobulins, which may protect their offspring against infection or severe disease. The time frame and duration after infection or vaccination, during which these immunoglobulins are detected in HM, as well as the major factors that influence their levels, have not been fully elucidated. This systematic review aimed to collect the existing literature and describe the immune response, specifically regarding the immunoglobulins in HM after COVID-19 disease or vaccination in non-immune women. We conducted a systematic search of PubMed and Scopus databases to identify studies published up until 19 March 2023. In total, 975 articles were screened, and out of which 75 were identified as being relevant and were finally included in this review. Infection by SARS-CoV-2 virus primarily induces an IgA immune response in HM, while vaccination predominantly elevates IgG levels. These immunoglobulins give HM a neutralizing capacity against SARS-CoV-2, highlighting the importance of breastfeeding during the pandemic. The mode of immune acquisition (infection or vaccination) and immunoglobulin levels in maternal serum are factors that seem to influence immunoglobulin levels in HM. Further studies are required to determine the impact of other factors, such as infection severity, lactation period, parity, maternal age and BMI on immunoglobulin level in HM.

## 1. Introduction

The global COVID-19 pandemic has resulted in more than 6.85 million deaths worldwide with about 0.1% of incidents occurring in neonates and children under 5 years [1,2]. The widespread availability of vaccines has played a crucial role in controlling transmission rates and reducing morbidity and mortality. The Center for Disease Control and Prevention (CDC) recommends vaccination for individuals aged 6 months and older, including neonates and non-vaccinated infants [3].

Younger or unvaccinated infants are defenseless against SARS-CoV-2 virus. Breastfeeding could be a protective factor against severe infection for these infants. Human milk (HM) contains various bioactive nutrients, such as immunoglobulins that block the penetration of microorganisms into the endothelium [4]. Initial concerns regarding the safety of breastfeeding during maternal infection led to previous recommendations for infected women to avoid breastfeeding. However, since June 23, 2020, the World Health Organization (WHO) strongly recommends breastfeeding, as the benefits outweigh the potential risks [5]. Recent studies indicate that maternal vaccination against SARS-CoV-2 virus reduces the risk of hospitalization in infants by approximately 60% [6].

SARS-CoV-2 virus is a single-stranded RNA virus, and its RNA is enveloped to a nucleocapsid. Its genome encodes four structural proteins: N(Nucleocapsid), M(Membrane), S(Spike) and E(Envelope) proteins [7]. The N protein is found in the virus core, and it forms complexes with viral RNA. It is also found in infected cells, so it is a common target for antigen tests [8,9]. The other three proteins are found in the viral envelope. The S protein interacts with the Angiotensin-converting enzyme 2(ACE2) receptor and mediates SARS-CoV-2 to be inserted into the host’s cells. The S protein consists of two subunits: the S1 subunit which contains an exposed receptor-binding domain (RBD), the part of the S protein that binds to the ACE2 receptor, and the S2 subunit for membrane fusion [10]. Tests that are used to evaluate the immune response after the vaccination target S protein or a subunit of it. Serology tests that are used in cases of COVID-19 disease, can target the N protein as well.

Nicolaidou V. et al., in a recent systematic review, reported that the HM of vaccinated lactating women contains neutralizing immunoglobulins against SARS-CoV-2 [11]. The presence of specific antibodies against the virus in the HM of vaccinated women has also been confirmed by another recent meta-analysis by Whited and Cervantes [12]. COVID-19 disease leads to an immune response in maternal organisms as well, and neutralizing antibodies are detected in their HM [13]. However, the duration that these antibodies remain in detectable levels, the time frame in which the immune response begins to wane, the factors that influence their levels in HM and the differences in the immune response between infected women and those who are vaccinated still remain unclear. We conducted a systematic review of the current literature from the beginning of the pandemic until March 19, 2023, in order to synthesize the current knowledge regarding the presence of antibodies against SARS-CoV-2 in HM after COVID-19 disease or vaccination, among non-previously immune pregnant or lactating women.

## 2. Materials and Methods

In order to perform this systematic review, we followed the Preferred Reporting Items for Systematic Reviews and Meta-Analyses guidelines’ (PRISMA) recommendation (presented as Supplementary Material) [14]. The systematic review was not registered in Prospero. We searched the Pubmed and Scopus databases from 1 December 2019 to 19 March 2023. We searched the existing literature only written in the English language.

The keywords used for the literature search were as follows: “SARS-CoV-2”, “Covid 19”, “novel coronavirus”, “Immunoglobulin*”, “antibody”, “IgG”, “IgA”, “secretory IgA”, “sIgA”, “immunological”, “immune system”, “immunogenicity”, “immunology”, “milk transferred antibody”, “breast milk”, “maternal milk”, “human milk”, “breastmilk”, “colostrum”, “breastfeeding”, “donor milk”, “lactating women” and “lactation”, combined with Boolean logical operators (AND, OR).

Additionally, we searched all of the references of the relevant studies and of previous corresponding systematic reviews in order to confirm the study saturation.

### 2.1. Study Eligibility Criteria

All selected studies examined the immunological response in the HM of pregnant or lactating women after COVID-19 disease or vaccination. After the duplicates were deleted, two investigators (M.D. and R.S.) independently checked the titles and abstracts of the retrieved papers, and consequently studied the full texts to decide which of them were eligible for the review. Any disagreements between the two researchers were analyzed and resolved by a third researcher (Z.I.).

Studies included in the present review met the following eligibility criteria: (1) women vaccinated against or infected by SARS-CoV-2 virus during pregnancy or lactating period were the study population, (2) there was no history of previously confirmed COVID-19 disease or vaccination, (3) current COVID-19 disease was confirmed via a PCR positive test, serology test or other laboratory method and (4) data on HM-specific immunoglobulins against SARS-CoV-2 were described in the studies that were included in the review as well. Irrelevant or non-original studies, case reports or studies with indecisive data were excluded from this review, as well as studies in any language other than English.

### 2.2. Data Extraction

The 2 researchers (M.D. and R.S.) separately studied the eligible studies and extracted useful data in an electronical database (Microsoft Excel). Complete information included the name of the first author, date of publication, country of origin, duration and population of the study, diagnosis method or vaccine type, time of HM collection, studied immunoglobulins and main outcomes or results of the study. Any disagreement between the two researchers was analyzed and resolved by a third researcher (Z.I.). Finally, the selected studies were examined again by another investigator (N.I) to check for eligibility and for duplication, and the extracted data were checked for their accuracy.

## 3. Results

**Figure 1:**
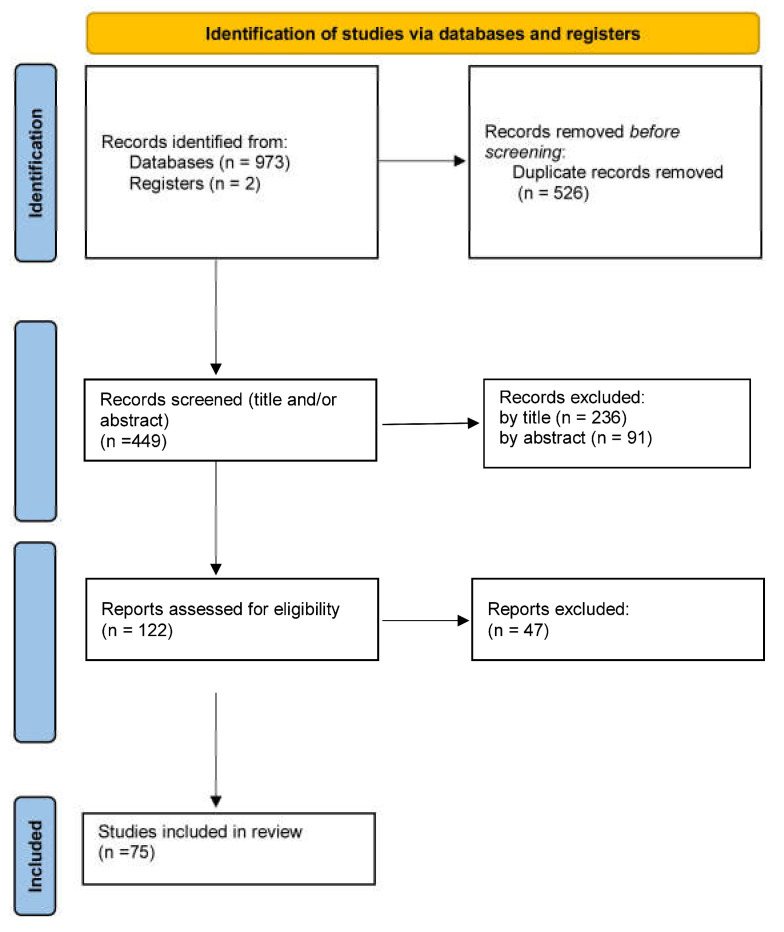
Flow chart of the study selection process.

A total of 975 articles were initially retrieved. After excluding duplicates and articles with titles and abstracts not related to our review object, 122 studies were selected for their full text to be studied. In these studies, the full text was comprehensively studied and 75 studies finally met the eligibility criteria. The searching and selection processes are depicted in a PRISMA graph (Figure 1).

Seventeen studies out of the total included women infected for the first time during pregnancy (Table 1) [9,10,11,12,13,14,15,16,17,18,19,20,21,22,23,24,25,26]. Another 17 studies examined women infected for the first time during lactation (Table 2) [27,28,29,30,31,32,33,34,35,36,37,38,39,40,41,42,43]. Six more studies included vaccinated pregnant women not previously immunized (Table 3) [22,25,27,29,44,45]. Finally, 40 studies examined vaccinated lactating women not previously immunized (Table 4) [22,25,32,38,42,46,47,48,49,50,51,52,53,54,55,56,57,58,59,60,61,62,63,64,65,66,67,68,69,70,71,72,73,74,75,76,77,78,79,80]. Ten studies included more than one participant group, and therefore they have been put into more than one table [22,25,27,29,32,38,42,44,46,47].

In total, 874 infected pregnant women, 537 infected lactating women, 196 vaccinated pregnant women and 1450 vaccinated lactating women were included in this systematic review. In the case of pregnant women, all samples were collected postpartum and most of them were collected within the 1st week postpartum. In some cases, samples were collected later, even 6 months after delivery in one study [27]. HM samples from lactating women were collected in variable time-points, with the longest being 6–10 months post infection or vaccination [43,51,54,56,62]. All studies detected specific immunoglobulins against SARS-CoV-2. Infection mainly induces the production of specific IgA antibodies [17,30,31,32,33,34,35,36,37,38,39,40,41,42,43,46,47,83,84,85,86], while vaccination mostly elicits the IgG response [22,25,27,29,32,38,42,44,46,47]. From the included studies, it seems that maternal age and BMI do not influence antibody titers. Yet, the general immune response of the mother and consequently the titers of some immunoglobulins in HM and maternal serum seem to correlate with the levels of other immunoglobulins in HM [16,21].

SARS-CoV-2 virus was not detected in HM samples in any study [18,20,24,30,36,38,40], and no study reported severe side effects after vaccination [42,53,63,65,66,72].

## 4. Discussion

Breastfeeding is the best dietary choice for infants [4]. HM contains specific immunoglobulins that are produced in response to exposure of the mother to pathogens. These immunoglobulins maintain their structural integrity in the infant’s stomach, bind to intestinal mucus and prevent pathogens from entering the bloodstream [89,90,91]. The presence of specific immunoglobulins against SARS-CoV-2 in the HM of infected or vaccinated mothers could potentially shield neonates and infants from future infections or even from severe disease.

### 4.1. Post Infection Immune Response

This systematic review affirms that specific immunoglobulins against SARS-CoV-2 virus were detected in the HΜ of women who were infected during pregnancy or lactation. These immunoglobulins were mainly IgA, and specifically secretory IgA antibodies, and less IgM and IgG were found [17,20,25,28,32,35,36,43,82], which is compatible with the known proportion of antibody isotypes in human milk [92]. They also primarily (80%) targeted the RBD domain of the S1 subunit [17].

#### 4.1.1. Anti-SARS-CoV-2 IgA Immunoglobulins

IgA titers in HM increase one week after infection, and these titers are even higher 2 weeks post COVID-19 disease [40]. Many studies indicate that they remain high in HM and detectable even 2–3 months after infection [27,31,34,35,38,39,40,42,43,83,85,86]. Pace RM et al. reported that IgA remained positive in 77% of 64 lactating women 2 months after infection [40]. Junker HG et al. found that HM conversion was observed after a median of 15 days and IgA levels peaked after 35 days. After 70 days, however, IgA was detectable only in 33% of HM samples [42]. Conti MG et al. reported that IgA was detectable in all HM samples of 28 lactating women even 2 months after delivery [23]. These women had been infected during pregnancy, which indicates that IgA may persist for a longer period of time. Indeed, other studies confirm their persistence in HM even 5–10 months post COVID-19 disease [34,39,43]. Fox A. et al. reported that all of the 28 tested women in their study had detectable IgA in HM 4–10 months after infection and 43% of them had even higher titers than what they had at 1 month after the infection [43]. The presence of specific IgA against SARS-CoV-2 in HM is optimal for infants. According to a recent cross-sectional study in Brazil, the titers of IgA in the HM of women infected during pregnancy were negatively correlated with the presence of clinical symptoms in their neonates [83].

Various factors influence anti-SARS-CoV-2 IgA levels in HM. The concentration of specific IgA immunoglobulins in HM is positively correlated with the levels of total IgA, IgM and IgG titers in HM [21]. Additionally, levels of specific IgG in maternal serum are also significantly correlated with IgA levels in HM [16]. However, this is not the case for IgA titers in serum. There is no reported association between IgA titers in HM and maternal serum [25,35,47]. This is logical as IgA in HM after natural infection is not of serum origin, but of muscular origin [93].

As for the time from infection, the data are conflicting. Some report a negative correlation with antibody titers [21,23], while others report a positive correlation [37]. Infection induces a humoral response and antibody titers begin to rise. After an unknown period of time, they reach a peak, and then they begin to wane over time [94]. Therefore, contradicting results in the studies may be due to different time-points of sample collection.

Additionally, not all types of antibodies have the same response over time. Bobik T.V. et al. tested sIgA against specific epitopes of SARS-CoV-2 virus (N protein, linear NTD, RBD-SD1 and RBD) and reported that the levels of sIgA against N-protein and against linear NTD and RBD-SD1 were higher in women that were infected during the third trimester compared to women infected during the first and second trimesters. Regarding sIgA against RBD, they found that their levels were similar, independent of the trimester of pregnancy when infection occurred [19]. No correlation between time of infection during pregnancy and anti-RBD antibodies was reported by Szczygioł, P. et al. either [28]. This indicates that this kind of antibody is more stable over time. Interestingly, Wachman EM et al. reported that anti-RBD IgA titers were not stable but significantly higher in women infected during the first or second trimester of pregnancy [84].

The severity of COVID-19 disease is another factor, but its impact on antibody titers has not yet been clarified. Pace, R.M et al. reported higher concentrations of antibodies in the HM of women with symptomatic COVID-19 disease than in the HM of asymptomatic women; yet, the difference was not significantly important [40]. In other relevant studies, no association between the two parameters has been found [28,83]. Probably, the severity of the disease has a positive impact on antibody levels. Many other studies on the general population have reported that antibody titers are higher in people with severe/moderate COVID-19 disease [94]. More studies are required to reach safe conclusions. From the existing data in the literature, no correlation has been found between IgA levels in HM and maternal age or infant gender [35,37].

#### 4.1.2. Anti-SARS-CoV-2 IgM Immunoglobulins

IgM immunoglobulins are the second most abundant antibodies in HM, and yet their titers are significantly lower than IgA titers. In a prospective study, Decenti EC detected anti-SARS-CoV-2 antibodies in only 7.5% of milk samples [26]. Specific IgM against 2 SARS-CoV-2 was mainly detected in samples collected 10–40 days after infection, and after that, their levels declined [20,21]. In a relevant study by Luo QQ et al., a positive correlation between IgM levels in HM and maternal serum was found [24]. The presence of IgM antibodies against infectious diseases in HM can provide passive immunity to infants, while simultaneously hindering the entry and transportation of viruses, such as HIV, to the infant [95,96]. Given these findings, it is plausible that breastfeeding by SARS-CoV-2-infected mothers offers postpartum protection to the infant through antibodies, reducing the risk of viral transmission.

#### 4.1.3. Anti-SARS-CoV-2 IgG Immunoglobulins

HM contains low titers of IgG immunoglobulins, and in some studies they were not even detectable [20,24]. Decenti, E. C. et al. studied HM samples from 141 women, and they detected IgG only in 3% of the study population [26]. Bauerl et al. reported low IgG titers in HM, with an increase in these titers from Day 40 to Day 205 after COVID-19 disease [21]. Pullen KM et al. detected IgG in a low concentration, but they did not observe any significant change in the titers over time (the mean time of sample collection was 66 days post infection). They also claimed that IgG was functionally attenuated compared to IgA and IgM [35]. In another 2 studies, low titers of IgG 0–3 months after delivery were reported [23,81]. Contrarily to the previous studies, Fox. A. et al. reported that they detected anti-S IgG antibodies in 75% of participants, with 13% of them being present in high titers [43]. The factors that influence their titers in HM are not all clear, but there is a positive correlation between IgG titers in HM and serum [29,35,47].

### 4.2. Post Vaccination Immune Response

The vaccination of pregnant or lactating women against SARS-CoV-2 also induces the secretion of specific anti-spike antibodies in HM. However, there are some differences in this immune response compared to the one after natural infection. First of all, vaccination mainly induces an IgG response and less of an IgA response [22,25,32,38,42,46,47,48,49,50,51,52,53,54,55,56,57,58,59,60,61,62,63,64,65,66,67,68,69,70,71,72,73,74,75,76,77,78,79]. After mRNA vaccination, a 10-fold and 100-fold increase in IgA and IgG titers was observed, respectively [47]. Vaccination induces no significant increase in secretory antibodies’ titers. IgA seems to be almost exclusively of systemic and not mucosal origin [55]. Pietrasanta et al. measured two subtypes of specific anti-S IgA antibodies, IgA1, which has systemic origin, and IgA2, which is mainly detected in mucosal secretions, and they observed that the antibodies were mainly IgA1 [53]. These differences in immune response are probably due to the intramuscular route of vaccine administration [50,67].

#### 4.2.1. Anti-SARS-CoV-2 IgG Immunoglobulins

IgG titers were detected in 87–100% of women post vaccination [50,62,65,66,72,77]. Only in one study was a moderate IgG immune response (43% of women) observed [73]. IgG titers in HM increase after each dose [38,47,50,52,55,63,67,71,74,75]. The peak of anti-S1-IgG titers occurs about 1–2 weeks after the 2nd dose and after that they wane [48,51,52,53,56,62,65,66,71]. A recent longitudinal study reported that IgG antibodies’ half lives in HM are about 2 months [45]. In other relevant studies, no significant difference in IgG titers was observed between 30 and 60 days post vaccination [57,80]. Even IgG levels wane over time, but remain in detectable levels 2 [57], 3 [38,53,72] and 6 months post vaccination [49,73]. Contrarily with total IgG levels, secretory IgG antibodies continuously increase even 6 months after the first dose of mRNA vaccines [54]. The main IgG subclasses in HM after 2 doses of mRNA-based vaccines are IgG1 and IgG3, which are the main subclasses of IgG that emerge after viral infections [45,97]. Interestingly, Agostinis C. et al., in a recent study, demonstrated that the presence of anti-S IgG in the HM of vaccinated lactating women is capable of activating in vitro the complement [80]. All of these data indicate that vaccinated mothers are capable of providing protective antibodies to their offspring for a long time after their primary vaccination.

As for the factors influencing antibody levels in HM, numerous studies have confirmed a significant positive correlation between IgG titers in HM and maternal serum [25,29,45,47,48,51,56,62,63,64,69,75,77,79,80]. A positive correlation between the lactation period and the total antibody titers was also reported in a study by Trofin F. et al. (lactating period between 3 and 36 months) [57]. However, this was not confirmed in other studies during the lactation period between 1.5 and 23 months [50,73]. A negative correlation was reported with parity [57], while no correlation was confirmed between IgG levels and maternal age or BMI [57,69,74].

#### 4.2.2. Anti-SARS-CoV-2 IgA Immunoglobulins

IgA immunoglobulins in detectable levels are present in 75–95% of vaccinated women at 2 weeks after the 2nd dose [42,63,65,66,74]. In a prospective study, though, detectable levels were present in just 36% of women post vaccination [73]. Yet, it is not clear if IgA rises mainly after the 1st dose, without any additional increase after the 2nd dose [38,47,63,67,73,74], or whether their titers present a biphasic model after vaccination [42,52,55,65,66,70,71]. Many studies indicate waning IgA titers about one month after the 1^st^ vaccine dose [44,49,51,56]. Contrarily, Juncker HG et al. reported that IgA titers peaked 2 weeks after the 1^st^ dose, and then they waned until the 2^nd^ dose, when they finally reached a second peak 5 days after vaccination [70]. Ricciardi et al. studied secretory IgA and they observed peak titers at 3 weeks after the 2nd dose, while their concentration significantly decreased at 6 months post vaccination [54]. Perez SE et al. detected IgA antibodies in about 50% and 25% of samples at 1 and 3 months post vaccination, respectively [62]. Finally, Narayanaswamy et al. observed no difference in anti-RBD IgA median titers before and after vaccination [50].

A positive correlation was found between IgA titers in HM and IgG titers in maternal serum [51,62,69,74]. A positive correlation may also exist between the IgA titers in HM and the IgA titers in maternal serum [25,47,64]; however, this finding is not supported by all studies [74]. The lactation period is also another factor which has an impact on IgA titers, and is not clearly defined by the included studies. A positive impact (lactation period between 3 and 36 months) [57] was reported in one study, while in others, either a negative (lactation period <18 months) [63] or no impact (lactation period 1.5–23 months for infants) [50] was reported. A negative correlation was found between antibody concentration and parity [57], and there was no correlation with maternal age [57,74].

#### 4.2.3. Anti-SARS-CoV-2 IgM immunoglobulins

Vaccination does not significantly influence IgM levels in HM. In the majority of studies, they are not even detectable [50,51,64,69,73,79], and in others they are just poorly detected [62,74].

### 4.3. Differences in Immune Response after Infection or Vaccination

Higher IgG titers were observed in the HM of vaccinated women compared to those who were previously infected with SARS-CoV-2 [22,25,32]. In the case of IgA, the data are ambiguous, with some studies reporting higher antibody titers after vaccination [27], some reporting lower [52] and some reporting no significant difference after vaccination or infection [42]. As has already been mentioned, infection mainly induces IgA and, to a lesser extent, the IgM immune response, while vaccination primarily stimulates the production of IgG antibodies. IgA and IgG are found mainly in secretory form and they are released by mammary tissue to HM. After a natural infection with SARS-CoV-2, B-cells stimulated in the lymphoid tissues of the respiratory tract migrate to the mammary gland and release secretory antibodies into HM [93]. Contrarily, post vaccination antiSARS-CoV-2 antibodies in HM are derived from maternal serum [11]. Secretory IgA antibodies are attached to the gastrointestinal tract of breastfeeding infants, where they bind with local microorganisms and block their penetration [4]. On the other hand, IgG antibodies produced post vaccination have been found to be capable of activating the complement. Therefore, infants can benefit from both types of antibodies in different ways [80].

As for the type of immunoglobulins, vaccination induces anti-spike protein antibodies. Infection, on the other hand, mainly induces the anti-N protein. A relative study by Bobik T.V. et al. reported significantly lower levels of the anti-RBD antibody compared to anti-N antibodies in the HM of lactating women infected during the third trimester [19]. L.Bode et al., in a recent study, reported that IgA antibodies in the HM of infected women are mainly directed against the N protein (about 43%) and less against the S protein (about 24%), and there was heterogeneity in the type and quantity of antibodies.

### 4.4. Differences in Immune Response According to Vaccine Type

No difference between detected antibody titers was described in women vaccinated with Moderna or Pfizer/BioNtech vaccines [32,52,57,63,68,88]. Only in one study of Gray KJ et al. were higher IgA titers detected in the Moderna group after the 2nd dose [67]. However, compared to the adenovirus-vectored vaccines, mRNA vaccines induce higher titers of antibodies [52,58,61]. Yang X. et al. reported that IgG and IgA levels were detectable in 86–100% and 52–71%, respectively, in mRNA-vaccinated women, and in 33–38% and 17–23%, respectively, in the adenovirus-vector-vaccinated women [58]. The Moderna vaccine also induces significantly higher titers of secretory antibodies compared with the rest of the vaccines [58]. Yet, six months post vaccination, no significant difference in antibody titers was observed among three types of vaccines [88].

### 4.5. Neutralizing Capacity

Although specific antibodies against SARS-CoV-2 are present in HM after COVID-19 disease or vaccination, it is essential to clarify whether these antibodies have neutralizing capacity. A neutralizing antibody binds with the viral surface and blocks its replication cycle, and so it protects the subject from subsequent infection [98]. Both infection [18,27,30,32,36,43,44,82] and vaccination [22,27,32,44,50,51,53,56,62,71,76,77] induce the production of neutralizing antibodies in HM, more intense, though, post infection [38,44].

In a study of 38 infected lactating women, no correlation between specific anti-SARS-CoV-2 antibody levels and the neutralization capacity of the HM was observed [31]. However, this is not in line with what other studies support. The neutralizing capacity against SARS-CoV-2 seems to be greater in the HM of infected women compared to the pre-pandemic controls, and it also seems to be positively correlated with antibody titers [18,27,30,32,36,43,44,82]. In a relevant study, 62% of the samples had neutralizing antibodies in vitro. Contrarily, the samples collected from the pre-pandemic controls had no neutralizing capacity [36]. Pace RM et al. reported that the HM neutralizing capacity post infection is significantly correlated with anti-RBD antibody levels [30].

Antibody titers also seem to influence the neutralizing capacity of the HM of vaccinated women [44,62]. As for variants, vaccination seems to be more beneficial against the Wuhan-Hu-1 strain and less against the Beta, Gamma and Delta variants. The binding capacity of antibodies is reduced by 30% to these strains [49]. In another study, less of a neutralizing capacity was reported against the Beta variant compared to D614G, Alpha and Gamma [50].

Our systematic review has some limitations. SARS-CoV-2 is a new virus. In the early stages of the pandemic, many studies were conducted with small sample sizes and lacked inclusion criteria. Furthermore, most of the tests used were homemade ELISA assays, which lacked standardization in terms of measurement units. Due to the great heterogeneity of the studies included, not only regarding the type of laboratory test, but also the differences in the timing of sampling between studies and study subjects (pregnant or lactating women), a meta-analysis was not deemed appropriate and instead we focused on presenting a systematic review of the available literature.

## 5. Conclusions

Infection with SARS-CoV-2 and vaccination against the virus elicit a maternal immune response in breastfeeding mothers with a short period of 1–2 weeks. Consequently, these mothers can transmit specific immunoglobulins with neutralizing capacity to their infants via HM. Therefore, it is recommended that breastfeeding is encouraged in mothers infected or vaccinated, as their HM can provide infants with specific antibodies even months after infection or vaccination.

Infection primarily induces an IgA-mediated immune response, while vaccination mainly elevates IgG immunoglobulins. Our data suggest that both IgA and IgG immunoglobulins contribute to the neutralizing capacity of HM, indicating clinical benefits for infants who receive HM from vaccinated or infected women. However, further studies are required to determine whether factors such as infection severity, lactation period, parity, maternal age and BMI have an impact on the levels of immunoglobulins in HM.

## Figures and Tables

**Table 1:** Characteristics of included studies referring to first-time-infected pregnant women.

Author (Year, Country)	Duration of Study	Pregnant Women (N)	Diagnostic Test	Time of Sample HM Collection	Studied Immunoglobulins	Antibody Measurement Method	Outcome/Results
Gao X. et al. (2020, China) [18]	20 days	12	PCR or serology test	Within 7 days postpartum	Anti-SARS-CoV-2 antibodies (IgM and IgG)	CLIA commercial kit/NAL	Neutralizing antibodies present in 3 samples of HM. SARS-CoV-2 virus was not detected in any sample.
Narayanaswamy V. et al. (2021, USA) [15]	5 months	15	PCR test	Within 48 hours postpartum	Anti-RBD antibodies (IgA, IgM and IgG)	Homemade ELISA	Present antibodies in colostrum (IgA in 73%, IgG in 73% and IgM in 33% of the samples).
Larcade R. et al. (2022, Argentina) [16]	2.5 months	58	PCR test	Within 96 h postpartum	Anti-RBD antibodies (IgA)	Homemade ELISA	Antibodies present in the HM of 87% of participants. No significant correlation with time of infection. Positive correlation between serum IgG titers and IgA titers in HM.
Bobik T.V. et al. (2021, Switzerland) [19]	NA	41	PCR test	Postpartum	Anti-S and anti-N antibodies (sIgA)	Homemade ELISA	Anti-RBD antibodies detected even in the HM of women infected during the first trimester.
Peng S. et al. (2020, China) [20]	NA	19	PCR test	Days 3,7,14,21, 28,42,56,70 postpartum	Anti-SARS-CoV-2 antibodies (IgM and IgG)	ELISA commercial kit	IgM present in the HM of 47% of participants. IgG was not detected in any sample. SARS-CoV-2 virus was not detected in any sample.
Bäuerl C. et al. (2021, Spain) [21]	8 months	60	PCR or serology test	Postpartum	Anti-RBD antibodies (IgA, IgM and IgG)	Homemade ELISA	Antibodies present in about 85% of samples. IgA titers remained stable in high titers over time. IgG titers were rising over time. Positive correlation between total IgA titers and anti-RBD IgA titers in HM.
Collier-ARY. et al. (2021, USA) [22]	1 year	22	PCR test	Postpartum	Anti-RBD antibodies (IgA and IgG)	Homemade ELISA	Antibodies present in HM.
Conti MG. et al. (2021, Italy) [23]	6 months	28	PCR test	2 days and 2 months postpartum	Anti-S antibodies (IgA and IgG)	ELISA commercial kit	IgA present in all samples. Higher titers in 48 h compared to 2 months post delivery. IgG titers remain low and stable over time.
Luo QQ. et al. (2021, China) [24]	43 days	4	PCR test	1 week postpartum	Anti-SARS-CoV-2 antibodies (IgM and IgG)	Homemade ELISA	IgM present in all samples of confirmed disease. Positive correlation between IgM titers in HM and in serum. IgG was not detected in any sample. SARS-CoV-2 virus was not detected in any sample.
Conti, M. G. et al. (2022, USA) [25]	2 months	28	PCR test	5 days and 2 months after infection	Anti-S antibodies (IgA and IgG)	ELISA commercial kit	IgA present in higher titers than IgG in HM. No correlation between IgA titers in HM and in serum.
Decenti, E. C. et al. (2022, Italy) [26]	16 months	141	PCR test	Postpartum	Anti-SARS-CoV-2 antibodies (IgM and IgG)	CLIA, ELISA, ECLIA, CMIA commercial kits	IgG present in 3.0% and IgM in 7.5% of HM.
Gu, Y. et al. (2022, Singapore) [81].	NA	8	PCR test	1–3 months postpartum	Anti-S and anti-RBD antibodies (IgA and IgG)	Homemade ELISA	Antibodies present in minimal levels 0–3 months postpartum.
Leung, H. Y. H. et al. (2022, China) [44]	NA	18	Laboratory-confirmed	3–192 days post infection (median = 45 days)	Anti-RBD antibodies (IgA)	Homemade ELISA/BA-Nabs	Antibody titers wane over time. Neutralizing antibodies present in HM. Positive correlation between antibody titers and binding capacity.
Martin-Vicente, M. et al. (2022, Spain) [82]	NA	46	PCR or serology test	Postpartum	Anti-S antibodies (IgA, IgM and IgG)	Homemade ELISA/NAA	Antibodies’ titers correlate positively with neutralizing capacity. IgA detected in higher titers than IgM and IgG.
Olearo, F. et al. (2022, Germany) [27]	6 months	9	PCR test	Monthly for 6 months postpartum	Anti-S antibodies (IgA) and anti-RBD antibodies (IgA, IgM and IgG)	Homemade ELISA/NAA	Presence of neutralizing antibodies in HM.
Szczygioł, P. et al. (2022, Poland) [28]	2.5 months	72	PCR test	1–229 days after infection (median 68)	Anti-RBD antibodies (IgA and IgG)	ELISA commercial kit	No correlation between antibody titers and severity of disease or time of infection.
Nir O. et al. (2022, Israel) [29]	2 months	11	PCR test	Postpartum	Anti-RBD antibodies (IgG)	Homemade ELISA	Positive correlation between IgG titers in HM and serum.
Fox, A. et al. (2022, USA) [43]	10 months	74	PCR test	4–6 weeks after infection and 4–10 months after infection	Anti-S antibodies (IgA and IgG)	Homemade ELISA/PNA	Detectable IgA in 89% of samples 4–6 weeks after infection. Detectable IgG in 75% of samples 4–6 weeks after infection. Detectable IgA even 4–10 months after infection. Increased neutralizing capacity of HM after infection.
Dutra LV. et al. (2023, Brazil) [83]	1 year	165	PCR test	1–2 days postpartum	Anti-S antibodies (IgA and IgG)	ELISA commercial kit	Detectable IgA in about 70% of participants. Negative correlation between IgA titers in colostrum and neonatal symptoms.
Wachman EM. et al. (2023, USA) [84]	1 year	31	PCR test	At delivery and 6 weeks postpartum	Anti-RBD and anti-N antibodies (IgA, IgM and IgG)	Homemade ELISA	Higher titers of anti-RBD IgA in the HM of women infected during first or second trimester. Positive correlation between IgG titer in maternal HM and serum.
Clabretto M. et al. (2023, Italy) [85]	8 months	12	PCR test	Postpartum	Anti-N antibodies (IgA) and anti-S antibodies (IgM and IgG)	CLIA and ELISA commercial kits	Presence of IgA in 66% of samples. Presence of IgM and IgG in no sample.

Anti-N antibodies = antibodies against nucleocapsid, anti-S antibodies = antibodies against Spike protein, anti-RBD = antibodies against receptor-binding domain, BA-Nabs = binding assay for screening neutralizing antibodies, CLIA = Chemiluminescent Immuno Assay, CMIA = Chemiluminescent Microparticle Immuno Assay, ECLIA = Electrochemiluminescent Immuno Assay, ELISA = enzyme-linked immunosorbent assay, IgA = Immunoglobulin A, IgG = Immunoglobulin G, IgM = Immunoglobulin M, NA = not acquired, NAAs = neutralizing antibody assays, PCR test = Polymerase chain reaction, PNA = Pseudovirus neutralization assay.

**Table 2:** Characteristics of included studies referring to first-time-infected lactating women.

Author (Year, Country)	Duration of Study	Lactating Women (N)	Diagnostic Test	Time of Sample HM Collection	Studied Immunoglobulins	Antibody Measurement Method	Outcome/Results
Pace R.M. et al. (2020, Moscow) [30]	NA	18	PCR test	12.0 ± 8.9 days after infection	Anti-SARS-CoV-2 antibodies (IgA and IgG)	Homemade ELISA/MNA	Antibodies present in HM samples. Positive correlation between anti-RBD antibodies’ titers and neutralizing capacity. SARS-CoV-2 virus was not detected in any sample.
van Keulen, BJ. (2021, Switzerland) [31]	ΝA	19	PCR test	5.9 (SD = 2.6) weeks after infection	Anti-S antibodies (IgA and IgG), anti-RDB and anti-N antibodies (IgA, IgM, IgG)	Homemade ELISA/PNA	Antibodies present in the HM of 83% of participants. No correlation between antibody titers and neutralizing capacity.
Demers-Mathieu V. (2021, USA) [32]	NA	10	PCR test	63 ± 40 days after infection	Anti-RBD antibodies (sIgA/IgA, sIgM/IgM and IgG)	Homemade ELISA/PNA	Antibodies present in HM. Higher inhibiting capacity against binding RBD to its receptor in women with previous COVID-19 infection compared to controls.
Demers-Mathieu V. et al. (2021, USA) [33]	NA	8	PCR test	2 months after infection	Anti-RBD antibodies (sIgA/IgA, sIgM/IgM and IgG)	Homemade ELISA	Antibodies present in HM.
Junker H.G. et al. (2021, the Netherlands) [34]	3.5 months	165	PCR test	8 weeks (mean time) after infection	Anti-SARS-CoV-2 antibodies (IgA)	Homemade ELISA	Detectable antibodies in HM even 10 months after infection.
Pullen K.M. (2021, USA) [35]	NA	20	PCR test	66 days (mean time) after infection	Anti-SARS-CoV-2 antibodies (IgA, IgM and IgG)	Systems serology	Antibodies present in HM. IgG detected in significantly lower titers. IgG functionally attenuated. Positive correlation between IgG titers in HM and serum. No correlation between IgA titers in HM and serum.
Pace, R.M. et al. (2021, USA) [36]	NA	18	PCR test	12 ± 8,9 days after infection	Anti-RBD, anti-S2, anti-N (IgA and IgG)	Homemade ELISA/MNA	IgA present in 76% of samples and IgG in 80% of samples. Total of 62% of samples had neutralization capacity. SARS-CoV-2 virus was not detected in any sample.
Demers-Mathieu V. et al. (2021, USA) [37]	NA	7	PCR test	3 ± 2 months after infection	Anti-RBD antibodies (sIgA/IgA, sIgM/IgM and IgG)	Homemade ELISA	Positive correlation between sIgA/IgA titers and time from infection. No correlation detected between antibody titers and maternal age, infant gender and severity of symptoms.
Young BE. et al. (2021, USA) [38]	5 months	47	PCR test	0, 3, 7, 10, 28 and 90 days after infection	Anti-RBD antibodies (IgA and IgG)	Homemade ELISA/MNA	IgA-dominant response. Detectable IgA in almost-stable titers even 3 months after infection. Increased neutralizing capacity of HM after infection. SARS-CoV-2 virus was not detected in any sample.
Juncker HG. et al. (2021, the Netherlands) [39]	3 months	29	PCR test	1,2,3,4 and 5 months after infection	Anti-S antibodies (IgA)	Homemade ELISA	Antibodies present even 5 months after infection. Not significant decrease in antibody titers over time.
Pace RM. et al. (2021, the Netherlands) [40]	8 months	64	PCR test	1,2,3,4 and 8 weeks after infection	Anti-RBD antibodies (IgA)	Homemade ELISA	Maximum IgA concentration was higher in symptomatic women. SARS-CoV-2 virus was not detected in any sample.
Demers-Mathieu V. et al. (2021, USA) [41]	NA	7	PCR test	47 +/− 24 days after infection	Anti-S1 or S2 subunit antibodies (sIgA/IgA, sIgM/IgM and IgG)	ELISA	Present anti-S2 IgA antibodies.
Juncker HG. et al. (2021, Switzerland) [42]	70 days	18	PCR test	Every 2 weeks after infection for at least 70 days	Anti-SARS-CON-2 antibodies (IgA)	Homemade ELISA	Detectable IgA antibodies even 70 days after infection.
Longueira, Y. et al. (2022, Argentina) [46]	12 months	11	PCR test	After infection	Anti-S antibodies (IgA and IgG)	ELISA commercial kit	Antibodies present in HM.
Narayanaswamy, V. et al. (2022, USA) [86]	4 months	30	PCR test	Every 3 days (for the 1st month after infection) and 4 months after infection	Anti-RBD antibodies (IgA, IgM and IgG)	Homemade ELISA/NAA	Detectable neutralizing antibodies. Detectable IgA and IgG in the majority of participants even 4 months after infection.
Wang, J. et al. (2022, USA) [47]	28 days	45	PCR test	0, 3, 10, 19 and 28 days after enrolled day (within 14 days from infection)	Anti-S and anti-N antibodies (IgA and IgG)	LUMINEX assay	Antibodies present in HM. Positive correlation between IgG titers in HM and serum. No correlation between IgA titers in HM and serum.
L. Bode. et al. (2022, USA) [87]	7 months	21	PCR test	Around infection	Anti-S, anti-RBD, anti-NTD and anti-N antibodies (IgA and IgG)	ECLIA	IgA was mainly against N protein and less against S protein. No difference was observed in IgG isotype. Heterogeneity in responses between participants.

Anti-N antibodies = antibodies against nucleocapsid, anti-NTD = antibodies against N-terminal domain, anti-S antibodies = antibodies against Spike protein, anti-RBD = antibodies against receptor-binding domain, CLIA = Chemiluminescent Immuno Assay, CMIA = Chemiluminescent Microparticle Immuno Assay, ECLIA = Electrochemiluminescent Immuno Assay, ELISA = enzyme-linked immunosorbent assay, IgA = Immunoglobulin A, IgG = Immunoglobulin G, IgM = Immunoglobulin M, MNA = microneutralizing assay, NA = not acquired, NAA, neutralizing antibody assay, PCR test = Polymerase chain reaction, PNA = Pseudovirus neutralization assay.

**Table 3:** Characteristics of included studies referring to vaccinated pregnant women not previously infected or vaccinated.

Author (Year, Country)	Duration of Study	Pregnant Women (N)	Vaccine Type	Time of Sample HM collection	Studied immunoglobulins	Antibody Measurement Method	Outcome/Results
Conti, M. G. et al. (2022, USA) [25]	NA	11	BNT162b2 2 doses	2 months after 2nd dose	Anti-S antibodies (IgA and IgG)	ELISA commercial kit	Higher IgG titers in vaccinated women compared to those who were infected. Positive correlation between IgG titers in HM and serum.
Leung, H. Y. H. et al. (2022, China) [44]	NA	8	COVID-19 vaccination	0–334 days post 1st dose (median = 71 days)	Anti-RBD antibodies (IgA)	ELISA commercial kit/BA-Nabs	Neutralizing antibodies present in HM. Antibody titers wane over time. Higher binding capacity after infection than vaccination. Positive correlation between antibody titers and binding capacity.
Olearo, F. et al. (2022, Germany) [27]	6 months	5	BNT162b2 2 doses	Monthly for 6 months postpartum	Anti-S antibodies (IgA) Anti-RBD antibodies (IgA, IgM and IgG)	Homemade ELISA	Higher titers of antibodies compared to recovered women.
Nir O. et al. (2022, Israel) [29]	2 months	64	BNT162b2 2 doses	Postpartum, within a few days	Anti-RBD antibodies (IgG)	Homemade ELISA	Positive correlation between IgG titers in HM and serum.
Collier-ARY. et al. (2021, USA) [22]	4 months	30	mRNA-1273/BNT162b2 2 doses	Close after each dose and 2–8 weeks after 2nd dose	Anti-RBD antibodies (IgA and IgG)	Homemade ELISA	Neutralizing antibodies present in HM.
Marshall N.E. et al. (2022, USA) [45]	NA	78	mRNA-1273/BNT162b2 2 doses	Between 0–12 months postpartum	Anti-RBD antibodies (IgG)	Homemade ELISA	Half life of IgG in HM is about 2 months. Positive correlation between IgG titers in HM and serum. IgG1 and IgG3 are present in higher titers compared to IgG4.

Anti-S antibodies = antibodies against Spike protein, anti-RBD = antibodies against receptor-binding domain, BA-Nabs = binding assay for screening neutralizing antibodies, ELISA = enzyme-linked immunosorbent assay, IgA = Immunoglobulin A, IgG = Immunoglobulin G, IgM = Immunoglobulin M, NA = not acquired.

**Table 4:** Characteristics of included studies referring to vaccinated lactating women not previously infected or vaccinated.

Author (Year, Country)	Duration of Study	Lactating Women (N)	Vaccine Type	Time of Sample HM Collection	Studied Immunoglobulins	Antibody Measurement Method	Outcome/Results
Demers-Mathieu V. (2021, USA) [32]	NA	19	mRNA-1273/BNT162b2 2 doses	37 ± 20 days after vaccination	Anti-RBD antibodies (sIgA/IgA, sIgM/IgM and IgG)	Homemade ELISA/PNA	Antibodies present in HM. Higher IgG titers in vaccinated compared to infected women. Higher neutralizing capacity in vaccinated women compared to controls.
Young BE. et al. (2021, USA) [38]	5 months	30	mRNA-1273/BNT162b2 2 doses	Prevaccination, 18 days after 1st dose, 18 and 90 days after 2nd dose	Anti-RBD antibodies (IgA and IgG)	Homemade ELISA/MNT	IgG-dominant response. Increased IgG titers after each dose. Declined IgG titers by 3 months. Increased IgA titers only after the first dose. Increased neutralization capacity after vaccination.
Juncker HG. et al. (2021, Switzerland) [42]	70 days	26	BNT162b2 2 doses	Prevaccination 3, 5, 7, 9, 11, 13, 15–17 days after 1st dose and 2nd dose and 70 days after 1st dose	Anti-S antibodies (IgA)	Homemade ELISA	Biphasic response of IgA antibodies. Detectable antibodies in 96% of participants after 2 doses. Detectable antibodies in about 40% of participants 70 days post vaccination. No significant difference between IgA titers in the HM of lactating women either infected or vaccinated. No severe adverse effect after vaccination.
Conti, M. G. et al. (2022, USA) [25]	NA	12	BNT162b2 2 doses	10 days after 2nd dose	Anti-S antibodies (IgA and IgG)	ELISA commercial kit	Higher IgG titers in vaccinated women compared to the infected group. Positive correlation between IgG titers in HM and serum.
Esteve-Palau, E. et al. (2022, Spain) [48]	6 months	33	BNT162b2 2 doses	2 weeks after 1st dose and 2,4,12 and 24 weeks after 2nd dose	Anti-S1 and anti-N antibodies (IgG)	Serology test, commercial kit	Peak anti-S1-IgG titers 2 weeks after 2nd dose. Positive correlation between IgG titers in HM and serum. IgG titers wane over a 6 month period.
Longueira, Y. et al. (2022, Argentina) [46]	12 months	27	Sputnik V, ChAdOx1-S or BBIBP-CorV	21 and 65 days after first and 21, 65 and 120 days after second dose (mean time)	Anti-SARS-CoV-2 antibodies (IgA and IgG)	ELISA commercial kit	2.8-fold increase in IgG titer after the 2nd dose. IgA remained constant between 1st and 2nd dose. 1.6-fold decrease in IgG titers, but stable levels of IgA over a 3-month period. Higher titer of IgG in participants who received adenoviral-based vaccines compared to those who received inactivated SARS-CoV-2 Sinopharm. No difference in IgA titers.
Low, J. M. et al. (2022, Singapore) [49]	6 weeks	46	BNT162b2 2 doses	Prevaccination 3–7 days and 4–6 weeks after 2nd dose	Anti-RBD antibodies (IgA)	Homemade ELISA	Peak titers of IgA observed 3–5 days after 2nd dose. Total of 30% reduction in binding capacity of antibodies in Beta, Gamma and Delta variants compared to Wuhan-Hu-1 strain.
Narayanaswamy, V. et al. (2022, USA) [50]	NA	27	mRNA-1273/BNT162b2 2 doses	Prevaccination after 1st and 2nd dose	Anti-RBD antibodies (IgA IgM and IgG)	ELISA commercial kit/V-PLEX commercial kit	IgG-dominant immune response. No increase in IgA titers after 2nd dose. No correlation between lactation stage and antibody titers.
Bender, J. M. et al. (2022, USA) [51]	10 months	10	mRNA-1273/BNT162b2 2 doses	Prevaccination 1, 3, 6 months after 1st dose	Anti-RBD antibodies (IgA, IgM and IgG)	Homemade ELISA/sVNT	Peak antibody titers one month after the 1st dose. No significant increase in IgM titers. Positive correlation between IgG titers in serum, and IgG and IgA titers in HM. Increased neutralizing capacity after vaccination.
Collier-ARY et al. (2021, USA) [22]	4 months	16	mRNA-1273/BNT162b2 2 doses	Close to each dose and 2–8 weeks after 2nd dose	Anti-RBD antibodies (IgA and IgG)	Homemade ELISA]	Neutralizing antibodies present in HM.
Pieri, M. et al. (2022, Switzerland) [55]	7 months	43	mRNA-1273/BNT162b2 ChAdOx1-S 2 doses	Prevaccination 1 day before the 2nd dose, 3 weeks after the 2nd	Anti-SARS-CoV-2 antibodies (sIgA/IgA and IgG)	ELISA commercial kit	Higher titers of IgG and IgA after the 2nd dose. Stable high antibody titers 3 weeks after the 2nd dose.
Pietrasanta, C. et al. (2022, Italy) [53]	90 days	24	BNT162b2 2 doses	Prevaccination before 2nd dose and 30, 60 and 90 days after 2nd dose	Anti-S and anti-RBD antibodies (IgA, IgA1, IgA2 and IgG)	Homemade ELISA	Vaccination induced IgG and IgA (mainly IgA1) immune responses. Antibodies waned over time, but they were still in detectable titers 90 days after vaccination. No severe adverse effect after vaccination.
Ricciardi, A. et al. (2022, Italy) [54]	2,5 months	18	BNT162b2 2 doses	Prevaccination at the 2nd dose, 3 weeks after 2nd dose and 6 months after 1st dose	Anti-S antibodies (sIgA and sIgG)	ELISA commercial kit	Increased sIgA titers after the 1st dose. Peak sIgA titers 3 weeks after the 2nd dose. Significant decrease in sIgA titers 6 months post vaccination. Peak sIgG titers 6 months after the first dose.
Agostinis C. et al. (2023, Italy) [80]	6 months	22	BNT162b2/ ChAdOx1-S 2 doses	Post vaccination (max 75 days post vaccination)	Anti-S antibodies (IgA and IgG)	Homemade ELISA	Range of antibody titers among participants. Positive correlation between IgG titers in serum and HM. No correlation between time of sample collection and antibody titers. Presence of IgG antibodies that can activate the complement.
Selma-Royo, M. et al. (2022, Spain) [52]	ΝA	75	mRNA-1273/BNT162b2/ ChAdOx1-S 2 doses	Prevaccination 1,2,3–4 weeks after 1st and 2nd dose In ChAdOx1-S group, a sample collected before 2nd dose	Anti-RBD antibodies (IgA and IgG)	Homemade ELISA	Peaked IgG titers 2 weeks after 2nd dose. Peaked IgA titers 1 week after the 1st dose for mRNA vaccines. IgA titers did not increase further after the 2nd dose. Higher IgG and IgA titers after vaccination with mRNA vaccines compared to those vaccinated with adeno-vectored vaccine. No significant difference observed between 2 mRNA vaccines.
Guida M. et al. (2021, Italy) [59]	NA	10	BNT162b2 2 doses	20 days after 1st dose and 7 days after 2nd dose	Anti-S antibodies (total)	ECLIA commercial kit	Higher antibody titers after 2nd dose. No correlation between total anti-S antibodies in HM and serum.
Stafford, L. et al. (2022, USA) [56]	12 months	8	mRNA-1273/BNT162b2 2 doses Ad.26.COV2.S 1 dose	Prevaccination 15–30 after 1st dose (for mRNA-based vaccines) and 7–30 days, 60–75 days, 90–105 days and 6 months after last dose	Anti-SARS-CoV-2 antibodies (IgA and IgG)	ELISA commercial kit/PVA	Antibody titers wane over time. Detectable levels 6 months post vaccination. More significant decline in IgG titers compared to IgA titers. Positive correlation between IgG levels in serum and HM 6 months post vaccination. Increased neutralizing capacity of HM after vaccination, especially 6 months post vaccination.
Valcare V. et al. (2021, USA) [60]	4 months	22	mRNA-1273/BNT162b2 2 doses	Prevaccination, post 1st and 2nd dose	Anti-S antibodies (IgA and IgG)	ELISA	Detectable antibodies in HM after vaccination.
Trofin, F. et al. (2022, Romania) [57].	7 months	28	mRNA -1273/BNT162b2 2 doses	30 and 60 days post 2nd dose	Anti-RBD antibodies (IgA and IgG)	ELISA commercial kit	No significant decrease in antibody levels 60 days post vaccination. Positive correlation of antibodies with lactation period. Negative correlation with child parity. No correlation between antibody titers and maternal age or vaccine type.
Wang, J. et al. (2022, USA) [47]	40 days	30	mRNA-1273/BNT162b2 2 doses	Prevaccination, 18 days after 1st and 2nd dose	Anti-Spike antibodies (IgA and IgG)	Homemade ELISA	Peak IgG titers after the 2nd dose. Peak IgA after the 1st dose. Total of 10-fold and 100-fold increase in IgA and IgG post vaccination, respectively. Detectable IgG titers even 187 days after the 2nd dose. Positive correlation between IgG titers in HM and serum. No correlation between IgA titers in HM and serum.
Yang, X. et al. (2022, USA and UK) [58]	NA	54	mRNA-1273/BNT162b2 2 doses ChAdOx1-S/Ad.26.COV2.S 2 or 1 dose	Prevaccination and 14 days or 21–35 (J&J) days post last dose	Anti-Spike antibodies (IgA and IgG)	Homemade ELISA	mRNA vaccines induce higher IgA antibody titers compared to adenovirus-vectored vaccines. Detectable IgG titers in 86–100% of mRNA-vaccinated women and in 33–38% of adenovirus-vector-vaccinated women. Detectable IgA in 52–71% of mRNA-vaccinated women and in 17–23% of adenovirus-vector-vaccinated women. Vaccination does not lead to significant increase in secretory antibody titers. (<50% of samples had specific secretory antibodies.) Moderna vaccine induces 2-fold higher levels of secretory antibodies compared with other vaccines.
Ramirez DSR et al. (2021, Spain) [69]	2 months	93	mRNA-1273/BNT162b2 2 doses	14 days post 2nd dose	Anti-RBD antibodies (IgA, IgM and IgG)	ELISA commercial kit/NAA commercial kit	Detectable IgG and IgA but not IgM. Positive correlation between IgG titers in HM and serum, positive correlation between IgA and IgG titers in HM. No correlation between antibody levels and maternal age and BMI.
Lechosa-Muñiz C. et al. (2021, Spain) [61]	1 month	110	mRNA-1273/BNT162b2 2 doses ChAdOx1-S 1 dose	30 days after the last dose	Anti-SARS-CoV-2 antibodies (IgA and IgG)	Homemade ELISA, CLIA commercial kit	Higher titers of IgA and IgG in HM after mRNA vaccine compared to adenovirus-vectored vaccine (the latter received only 1 dose).
Gray KJ. et al. (2021, USA) [67]	NA	31	mRNA-1273/BNT162b2 2 doses	At time of 1st and 2nd dose, 2–5.5 weeks post 2nd dose	Anti-S and anti-RBD antibodies (IgA, IgM and IgG)	Homemade ELISA/LUMINEX	Robust IgG titers, but not IgA and IgM titers after the 2nd dose. Higher IgA titers detected to Moderna group after the 2nd dose.
Perez S.E. et al. (2021, USA) [62]	14 months	27	mRNA-1273/BNT162b2 2 doses	Prevaccination, 1, 3 and 6 months post vaccination	Anti-SARS-CoV-2 antibodies (IgA, IgM and IgG)	Homemade ELISA/sVNT commercial kit	Detectable IgG in all samples, IgA in about 1/2 of the samples and IgM in about 1/4 of samples. IgG reached their peak levels 1 month post vaccination. Detectable IgG titers even 6 months post vaccination. Detectable IgA and IgM titers even 3 months post vaccination. Positive correlation between IgG and IgA titers in HM and IgG titers in serum. Neutralizing capacity of HM even at 6 months post vaccination. Neutralizing capacity correlates positively with IgG titers.
Golan Y. et al. (2021, USA) [63]	NA	48	mRNA-1273/BNT162b2 2 doses	Prevaccination before 2nd dose and 4–10 weeks after 2nd dose	Anti-RBD antibodies (IgA and IgG)	Homemade ELISA	Higher IgA titers after the 1st dose. No further increase after the 2nd dose. Detectable IgA titers in 75% of participants. IgG titers increase after 1st dose, with further increase in their titers after 2nd dose. No significant difference in the immune response between mRNA-based vaccines. Positive correlation between IgG titers in HM and maternal serum. No severe adverse effect after vaccination.
Scaggs Huang F. et al. (2021, USA) [79]	3 months	98	mRNA-1273/BNT162b2 2 doses	14 days post 2nd dose	Anti-S1 (IgA, IgM and IgG) and anti-N antibodies (IgG)	Serology test/NAA	Anti-S1 IgG and IgA antibodies present in HM. IgM antibodies were not detectable in any sample. Positive correlation between IgG titers in serum and HM.
Jakuszko K. et al. (2021, Switzerland) [64]	3 months	28	BNT162b2 2 doses	8 ± 1, 22 ± 2, 29 ± 3 and 43 ± 4 after 1st dose	Anti-Spike antibodies (IgA, IgM and IgG)	ELISA commercial kit	Peak antibody titers 7 ± 3 days after 1st dose. Higher IgA titers in the HM of serum-positive IgA women compared to serum-negative ones. No IgM was detectable in any sample. Positive correlation between IgG titers in HM and serum.
Schwartz A. et al. (2021, Israel) [77]	5 months	61	BNT162b2 2 doses	Post vaccination	Anti-RBD antibodies (IgA and IgG)	ELISA	IgG antibodies were detectable in all samples. Positive correlation between IgG titers in serum and HM. Neutralizing antibodies were detected in about 40% of samples. Secretory IgA was detected in 15% of samples.
Perl SH. et al. (2021, Israel) [65]	8 weeks	84	BNT162b2 2 doses	Prevaccination 2, 3, 4, 5 and 6 weeks post 1st dose	Anti-SARS-CoV-2 antibodies (IgA and IgG)	CLIA commercial kit	Detectable IgA titers in 61.8% and 86.1% of participants at 2 and 4 weeks after the 1st dose, respectively. Increased IgA titers after the 1st and 2nd dose. Detectable IgA titers 6 weeks after the 1st dose. IgG titers increased 1 week after the 2nd dose. IgG was detectable in 97% of samples 5 and 6 weeks after the 1st dose. No severe adverse effects after vaccination.
Yeo KT. et al. (2021, Singapore) [76]	4,5 months	34	BNT162b2 2 doses	Prevaccination 1, 3, 7, 14 and 21 days after 1st and 2nd dose	Anti-RBD antibodies (IgA, IgM and IgG)	Homemade ELISA/s-VNA commercial kit	Neutralizing antibodies in HM increased after the 2nd dose. Neutralizing antibodies in HM were detectable even 3 weeks after 2nd dose. Dominance of IgG1 response post vaccination.
Low JM. et al. (2021, Singapore) [66]	NA	14	BNT162b2 2 doses	Prevaccination 1–3 and 7–10 days post 1st dose, 3–7 days and 4–6 weeks post 2nd dose	Anti-Spike and anti-RBD antibodies (IgA and IgG)	Homemade ELISA	Presence of IgA antibodies in 93% of participants. Higher ΙgA titers 3–7 days after the 2nd dose. IgA titers waned 4–6 weeks after the 2nd dose. IgG antibodies detected in all samples. IgG titers increased after the 2nd dose. Stable high IgG titers even 4–6 weeks after the 2nd dose. No severe adverse effects in participants.
Baird JK. et al. (2021, USA) [68]	NA	7	mRNA-1273/BNT162b2 2 doses	Prevaccination 1, 4, 7 and 14 days post 1st and 2nd dose and 1 day before 2nd dose	Anti-S and anti-RBD antibodies (IgA and IgG)	Homemade ELISA	Vaccination mainly induced IgG response. No significant difference between 2 vaccines. Antibodies present even 80 days after vaccination in 1 sample collected.
Juncker HG. et al. (2021, the Netherlands) [70]	1 month	20	BNT162b2 2 doses	Prevaccination 3, 5, 7, 9, 11, 13 and 15–17 days after 1st and 2nd doses and 1 day before 2nd dose	Anti-SARS-CoV-2 antibodies (IgA)	Homemade ELISA	Increased IgA titers 5–7 days after the 1st dose. IgA titers decreased by 50% between Day 15 and 21. IgA titers increased by 1.3 times after the 2nd dose compared to the 1st one.
Rosenberg-Friedman M. et al. (2021, Israel) [71]	NA	10	BNT162b2 2 doses	7 and 14 days post 1st and 2nd doses	Anti-S and anti-RBD antibodies (IgA and IgG)	Homemade ELISA	IgG titers peaked 7 days after the 2nd dose. IgG titers remained high on Day 14 after the 2nd dose. Increased IgA titers 7 days post the 2nd dose. Increased neutralizing capacity observed after vaccination.
Scrimin F. et al. (2022, Switzerland) [72]	6 months	40	mRNA-1273/BNT162b2 2 doses ChAdOx1-S 2 or 1 dose	20–30 days, 1–2 months or 3–4 months post 2nd dose	Anti-SARS-CoV-2 antibodies (IgA and IgG)	ELISA commercial kit/NAA	IgG antibodies detected in all samples. IgG present even 4 months after the 2nd dose. No severe adverse effects in participants.
Marshall N.E. et al. (2022, USA) [45]	NA	28	mRNA-1273/BNT162b2 2 doses	Between 0–12 months postpartum	Anti-RBD antibodies (IgG)	Homemade ELISA	Half life of IgG in HM is about 2 months. Positive correlation between IgG titers in HM and serum. IgG1 and IgG3 are present in higher titers compared to IgG4 antibodies.
Esteve-Palau E. et al. (2021, Spain) [75]	NA	33	mRNA -based BNT162b2 2 doses	2 weeks after 1st dose and 2 and 4 weeks after 2nd dose	Anti-S antibodies (IgG)	Serology test	Increased IgG titers after 2nd dose. Positive correlation between serum and HM IgG titers.
Gonçalves J. et al. (2021, Portugal) [74]	7 months	23	mRNA-1273/BNT162b2 2 doses	Prevaccination, a median of 10 days after 1st and 2nd dose	Anti-S antibodies (sIgA/IgA, IgM and IgG)	Homemade ELISA	Antibodies present in the HM of 96% of participants. Increased IgG titers after the 2nd dose. Increased IgA titers after the 1st dose. Poorly increased IgM antibodies after vaccination. Positive correlation between IgA and IgG titers in HM. Positive correlation between IgA titers in serum and HM. Antibody titers were not correlated with maternal age.
Lechosa-Muniz C. et al. (2023, Spain) [88]	6 months	62	mRNA-1273/BNT162b2 2 doses ChAdOx1-S 1 dose	6 months post vaccination	Anti-S antibodies (IgA and IgG)	Homemade ELISA/CLIA commercial kit	No significant difference in antibody levels according to vaccine type.
Charepe N. et al. (2021, Portugal) [73]	2 months	14	BNT162b2 2 doses	1–3 weeks post 1st and 2nd dose	Anti-S antibodies (IgA, IgM and IgG)	Homemade ELISA	Increased IgG titers after the 2nd dose. IgA peaked after the 1st dose. No IgM response was observed.
Kelly JC. et al. (2021, USA) [78]	NA	5	BNT162b2 2 doses	Prevaccination within 24 h after 1st dose and a week post vaccination	Anti-S antibodies (IgA and IgG)	ELISA	Anti-S antibodies were present in HM.

Anti-S antibodies = antibodies against Spike protein, anti-RBD = antibodies against receptor-binding domain, CLIA = Chemiluminescent Immuno Assay, ELISA = enzyme-linked immunosorbent assay, IgA = Immunoglobulin A, IgG = Immunoglobulin G, IgM = Immunoglobulin M, MNA = microneutralizing assay, NA = not acquired, NAA, neutralizing antibody assay, PCR test = Polymerase chain reaction, PNA = Pseudovirus neutralization assay, sVNT = surrogate virus neutralization assay.

## Data Availability

Data are contained within the article.

## Supplementary Materials

The following supporting information can be downloaded at: https://www.mdpi.com/article/10.3390/nu15081905/s1, Supplement: PRISMA 2020 check list.
